# Transscleral Sustained Vasohibin-1 Delivery by a Novel Device Suppressed Experimentally-Induced Choroidal Neovascularization

**DOI:** 10.1371/journal.pone.0058580

**Published:** 2013-03-05

**Authors:** Hideyuki Onami, Nobuhiro Nagai, Hirokazu Kaji, Matsuhiko Nishizawa, Yasufumi Sato, Noriko Osumi, Toru Nakazawa, Toshiaki Abe

**Affiliations:** 1 Division of Clinical Cell Therapy, United Centers for Advanced Research and Translational Medicine (ART), Tohoku University Graduate School of Medicine, Sendai, Japan; 2 Department of Ophthalmology, Tohoku University Graduate School of Medicine, Sendai, Japan; 3 Department of Bioengineering and Robotics, Graduate School of Engineering, Tohoku University, Sendai, Japan; 4 Department of Vascular Biology, Institute of Development, Aging and Cancer, Tohoku University, Sendai, Japan; 5 Division of Developmental Neuroscience, United Centers for Advanced Research and Translational Medicine (ART), Tohoku University Graduate School of Medicine, Sendai, Japan; Eye Hospital, Charité, Germany

## Abstract

We established a sustained vasohibin-1 (a 42-kDa protein), delivery device by a novel method using photopolymerization of a mixture of polyethylene glycol dimethacrylate, triethylene glycol dimethacrylate, and collagen microparticles. We evaluated its effects in a model of rat laser-induced choroidal neovascularization (CNV) using a transscleral approach. We used variable concentrations of vasohibin-1 in the devices, and used an enzyme-linked immunosorbent assay and Western blotting to measure the released vasohibin-1 (0.31 nM/day when using the 10 μM vasohibin-1 delivery device [10VDD]). The released vasohibin-1 showed suppression activity comparable to native effects when evaluated using endothelial tube formation. We also used pelletized vasohibin-1 and fluorescein isothiocyanate-labeled 40 kDa dextran as controls. Strong fluorescein staining was observed on the sclera when the device was used for drug delivery, whereas pellet use produced strong staining in the conjunctiva and surrounding tissue, but not on the sclera. Vasohibin-1 was found in the sclera, choroid, retinal pigment epithelium (RPE), and neural retina after device implantation. Stronger immunoreactivity at the RPE and ganglion cell layers was observed than in other retinal regions. Significantly lower fluorescein angiography (FA) scores and smaller CNV areas in the flat mounts of RPE-choroid-sclera were observed for the 10VDD, VDD (1 μM vasohibin-1 delivery device), and vasohibin-1 intravitreal direct injection (0.24 μM) groups when compared to the pellet, non-vasohibin-1 delivery device, and intravitreal vehicle injection groups. Choroidal neovascularization can be treated with transscleral sustained protein delivery using our novel device. We offer a safer sustained protein release for treatment of retinal disease using the transscleral approach.

## Introduction

Age-related macular degeneration (AMD) is a well-known sight-threatening disease in developed countries [1]. Although many treatment regimens have been used to treat AMD [2]–[6], intravitreal injection of anti-vascular endothelial growth factor (VEGF) produced lesion improvement and better visual acuity in some patients [7], [8]. However, intra-vitreal injection of anti-VEGF also produced irritation, infection, and other adverse side effects [9]. Further, that treatment required repeated injections, usually occurring once a month [7], [8]. Thus, other types of drugs or drug delivery systems (DDSs) need to be developed to treat AMD.

Eye drops and systemic drug administration are unsuitable for retinal diseases if the physician is looking for effective drug penetration into the eye, especially for macular diseases such as AMD [10], [11]. Although drug delivery device implantation into the vitreous showed effective delivery of drug to the retina, these treatments may cause severe side effects, such as infection, vitreous hemorrhage, or retinal detachment [12]–[14]. Drug delivery using viral vectors has been attempted for treatment of devastating retinal diseases [15]; however, this method may induce immune cell or humoral responses [16], [17].

Subconjunctival drug delivery is less invasive than intravitreal drug injection and can deliver more drug than seen with eye drops or systemic administration [10], [11]. There are published data investigating clinical use of subconjunctival drug administration [18], [19]. Thus, the subconjunctival route may be an attractive method for drug delivery to the retina. The major difficulties with subconjunctival DDS are uncontrollable release of the target drug [20], as well as an unknown drug delivery route and mechanism to reach the retina [20], [21]. Sustained release, with no drug bolus effect, would be required to reduce side effects [22], [23].

We previously reported our results of the use of a novel drug delivery device placed on the sclera that we thought would be an effective tool in treating retinal diseases [24]. The device consisted of a drug-releasing semi-permeable membrane and impermeable membranes acting as the drug reservoir. Because of the non-biodegradable and one-way release nature of the device, we could achieve sustained release of the drug to the retina. We examined the effects of this device using a laser-induced choroidal neovascularization (CNV) model in rats.

Anti-VEGF antibody is a well-known treatment agent in CNV therapy, but suppression of VEGF function may induce many harmful effects in physiological function [25]. We selected vasohibin-1 for the loading drug in the device in this study because of its well-known anti-angiogenic activity [26], [27]. Vasohibin-1 is a 42-kDa polypeptide, a VEGF-inducible molecule expressed by cultured human endothelial cells (ECs) [26]. Vasohibin-1 inhibits the formation of EC networks *in vitro*, corneal neovascularization *in vitro* [26], retinal neovascularization in a mouse model of oxygen-induced ischemic retinopathy [27], and laser-induced mouse [25] and monkey CNV [28]. Each of the *in vivo* studies treated the tissue by direct intravitreal injection of vasohibin-1.

**Figure 1:**
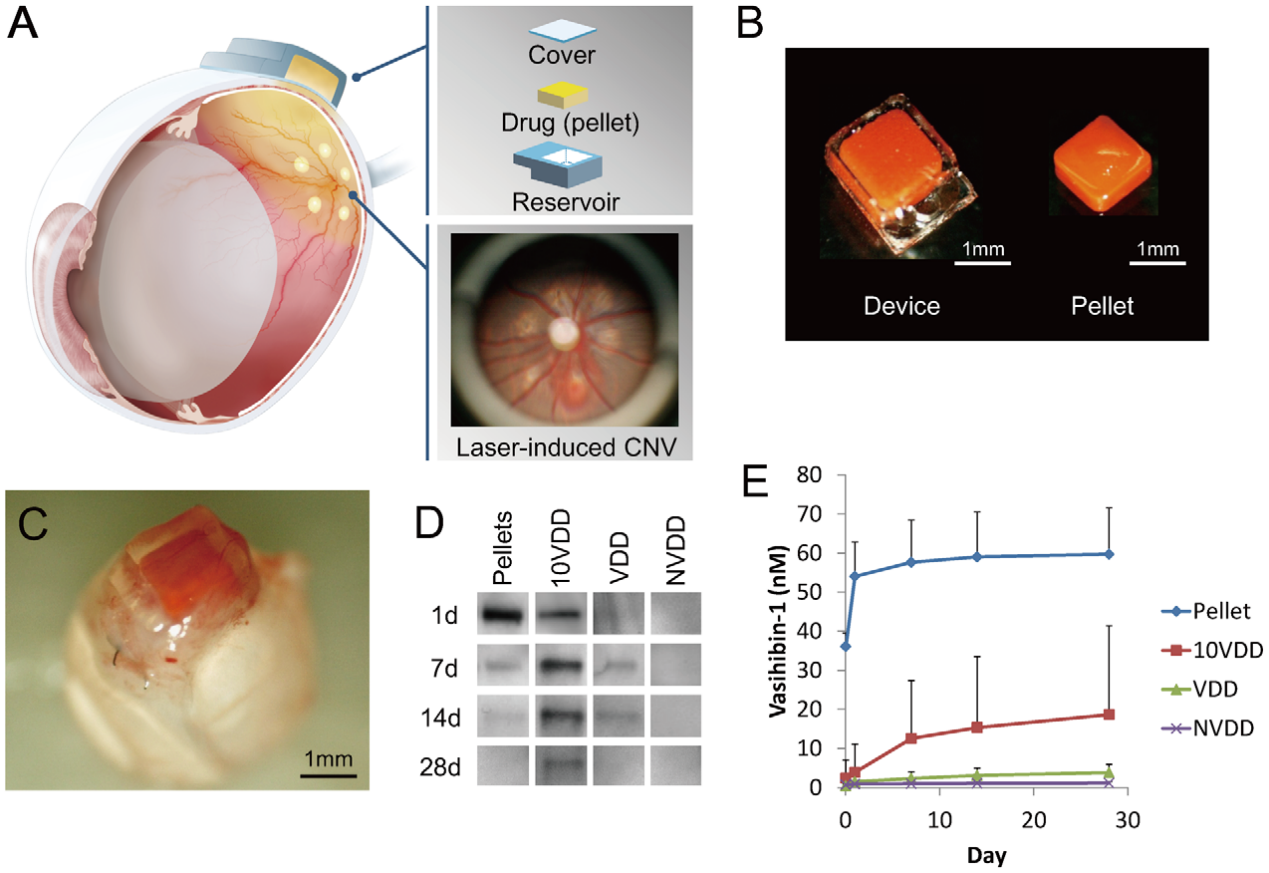
Device and vasohibin-1 release. (A) Schematic image of transscleral sustained vasohibin-1 delivery. We evaluated its effects via transscleral approach for rat laser-induced choroidal neovascularization (CNV). The device consists of a drug pelletized with PEGDM, a reservoir made of TEGDM, and a controlled-release membrane made of PEGDM that contains collagen microparticles. (B) Photograph showing a drug pellet and the delivery device containing a drug pellet. (C) Image of a device placed on the sclera of a rat eye at 3 days after implantation. The amount of vasohibin-1 in the PBS was measured at 1, 7, 14, and 28 days after starting incubation. The representative results of western blotting and the result of ELISA are shown in (D) and (E), respectively. We collected the samples at only the given time points and replaced only the equal volume of PBS. The released vasohibin-1 amounts accumulated for 6, 7, and 14 days. [The pellet samples collected at Day 1 (shown as 1d) were diluted five times due to their concentration before they were evaluated by western blotting]. NVDD: non-vasohibin-1 (vehicle) delivery device, VDD: 1 μM vasohibin-1 delivery device, 10VDD: 10 μM vasohibin-1 delivery device, Pellets: vasohibin-1 pelletized at the same concentration of 10VDD (without reservoir and cover).

Here we shall show that continuous trans-scleral vasohibin-1 delivery by the device can suppress laser-induced CNV in rat eyes (Fig. 1A) as well as that by intravitreal injection. This technique and device may hold promise for safer and more effective treatment of patients with AMD.

## Methods

### Vasohibin-1 and Device Preparation

Vasohibin-1 was purified as reported previously [25]. For the preparation of the vasohibin-1 formulation, an 80-μL volume of vasohibin-1 (either 1.25 or 12.5 μM) in vehicle (phosphate buffered saline [PBS] control) was mixed with 20 μL of polyethylene glycol dimethacrylate (PEGDM), then underwent UV curing at an intensity of 11.5 mJ/cm^2^ (Lightningcure LCS; Hamamatsu Photonics, Hamamatsu City, Japan) for 3 minutes.

The devices consisted of a semi-permeable drug-releasing membrane and an impermeable reservoir (Fig. 1A, 1B), as we reported previously [24]. The loaded vasohibin-1 doses included vehicle only (identified as NVDD), 1 μM vasohibin-1 (VDD), and 10 μM vasohibin-1 (10VDD), with a total volume of 1.5 μL in each device. The size of the device was 2 mm×2 mm wide ×1 mm high (drug-releasing surface area; 1.5 mm×1.5 mm  = 2.25 mm^2^) for the rat experiments (Fig. 1B, device) and 4 mm×4 mm×1.5 mm (drug-releasing surface area; 3.5 mm×3.5 mm  = 12.25 mm^2^) for the vasohibin-1 releasing *in vitro* assay. The release amount from the transplanted device was small and it was very difficult to detect released vasohibin-1 by the standard ELISA technique, so we decided to use a larger device for the ELISA procedure. As a control, we used pelletized vasohibin-1 without the reservoir and permeable membrane (Fig. 1B, pellet). The concentration of pelletized vasohibin-1 was adjusted to be the same concentration as that of the 10VDD (10 μM vasohibin-1). The total amount of vasohibin-1 released from the 10VDD device during the 2-week *in vivo* experiment was aimed to be equivalent to that of the intravitreal vasohibin-1 injection. A FITC-labeled 40 kDa dextran-loaded device (FD40DD) was also used for monitoring the position of the implanted device.

### *In Vitro* Experiments

#### *1 In Vitro* Release Assay, Enzyme-linked Immunosorbent Assay, and Western Blotting

The devices loaded with vasohibin-1 were placed in the wells of a 24-well culture plate filled with 200 μL PBS at 37°C. Aliquots (200 μL) of the buffer in each well were collected at Days 1, 7, 14, and 28 during change-out of old buffer for new buffer solution. The collected samples were considered to include only protein for vasohibin-1. We then determined the amount of vasohibin-1 in the buffer using an enzyme-linked immunosorbent assay (ELISA) [29] and western blotting [30]. The intensity of the color of the ELISA reaction products was measured with a microplate reader (MAXline; Molecular Devices Corporation, Sunnyvale, CA, USA). The measurements were made in duplicate, and the mean value was used for comparisons. The 50-µL collected samples and 100 fmol of recombinant vasohibin-1 (positive control) were loaded, separated by sodium dodecyl sulfate-polyacrylamide gel electrophoresis (SDS-PAGE) on a 10% separating gel, and transferred to nitrocellulose membranes for western blotting. The membranes were blocked for 1 hour at room temperature with 5% ECL blocking agent (GE Healthcare Biosciences, Pittsburgh, PA, USA), and then incubated overnight at 4°C in PBS containing 0.05% Tween 20 (T-PBS), 2.5% skim milk, and 1 μg/mL horseradish peroxidase-conjugated anti–vasohibin-1 monoclonal antibody. The membrane filters were washed 3 times with T-TBS and the blots were detected using an enhanced chemiluminescence method (ECL Western Blotting Detection Kit; Amersham Biosciences, Piscataway, NJ, USA). The results were visualized using an imaging system (ImageQuant LAS-1000; GE Healthcare Biosciences).

#### 2 Endothelial Tube Formation

Endothelial tube formation was assessed with normal human umbilical vein endothelial cells (HUVECs) (Takara Bio; Otsu, Japan) co-cultured on neonatal normal human dermal fibroblasts (NHDF, Takara Bio) layer using anti-human CD31 immunostaining, as reported previously [28]. Two nM vascular endothelial growth factor (VEGF, Wako; Tokyo, Japan) was then added to the endothelial cell growth medium (EGM, Takara Bio) containing no vasohibin-1 (control), and 0.2, 2, or 10 nM vasohibin-1, respectively. VEGF (2 nM) and samples of vasohibin-1 released from the vasohibin-1-loaded device over 3 hours at 37°C were used to examine released vasohibin-1 activity. We collected the released vasohibin-1 from the pellet and used it at a concentration of 0.56 nM (as measured by ELISA). On Day 3, the cells were fixed and stained using an anti-human CD31 immunostaining kit (Kurabo; Tokyo, Japan) according to the manufacturer's instructions. The number of stained HUVECs was determined using a computerized system (Kurabo Angiogenesis Image Analyzer program; Kurabo).

### *In Vivo* CNV Experiments

#### 1 Animals

The procedures used in the animal experiments followed the guidelines of the Association for Research in Vision and Ophthalmology Statement for the Use of Animals in Ophthalmic and Vision Research, and they were approved by the Animal Care Committee of Tohoku University Graduate School of Medicine (Permit Number: 2011–136). Twenty Sprague-Dawley (SD) rats (Experiments 1 and 2) and 36 Brown Norway (BN) rats (Experiment 3) weighing between 250 and 300 g were used (Table 1). All animals were followed up to 2 weeks after device transplantation and/or laser burn. We examined the effects of devices either at 1 week or 2 weeks for FA evaluation and 2 weeks for flat-mount evaluation. Macro examination was performed at 1 and 2 weeks after the device transplantation. For all procedures, the rats were anesthetized with an intramuscular injection of ketamine hydrochloride (35 mg/kg) and xylazine hydrochloride (5 mg/kg), and the animals' pupils were dilated with topical 2.5% phenylephrine and 1% tropicamide. Oxybuprocaine hydrochloride (0.4%) was also used for local anesthesia. In all *in vivo* experiments, the animal's left eye was used as a control.

**Table 1:** In Vivo Study Demographics.

Number of animals	Strain	Treatment	Methods	Position of implant
**Experiment 1**				
4	SD	Untreated	FD40DD	Sclera
4	SD	Untreated	FD40 Pellet	Sclera
**Experiment 2**				
4	SD	Untreated	NVDD	Sclera
4	SD	Untreated	10VDD	Sclera
4	SD	Untreated	Pellet	Sclera
**Experiment 3**				
6	BD	CNV	NVDD	Sclera
6	BD	CNV	VDD	Sclera
6	BD	CNV	10VDD	Sclera
6	BD	CNV	Pellet	Sclera
6	BD	CNV	Vehicle	Vitreous
6	BD	CNV	Vasohibin-1	Vitreous

SD: Sprague-Dawley rats, BN: Brown Norway rats, CNV: choroidal neovascularization, NVDD: non-vasohibin-1 delivery device, 10VDD: 10 μM vasohibin-1 delivery device.

#### 2 Implantation of VDDs, Pellets, and Intravitreal Vasohibin-1 Injection

Devices were implanted subconjunctively in the right eyes of the rats (Table 1). A 4-mm long conjunctival incision was made along the limbus in the upper temporal position. The devices were inserted into the subconjunctival space using forceps, with the drug-releasing surface facing the sclera. The device was placed between the optic disc and the equator, in the posterior quadrant, using no suture to anchor it into place. The conjunctival incision was closed with 9–0 silk and antibiotic ointment was applied to the eyes. Vasohibin-1 protein (0.24 μM) was injected using a 10-μL glass syringe (Hamilton; Reno, NV) 4 days after the experimental CNV procedure. The left eyes were used as untreated controls.

The rats were anesthetized, pupils were dilated, and a fundus examination was performed immediately after the surgery.

### Experiment 1: Monitoring the Implanted Devices and Pellets

To monitor the device and drug release, fluorescein isothiocyanate (FITC) dextran (FD40; Sigma-Aldrich) pelletized with PEGDM was prepared and used as a control drug. The FD40 was dissolved in PBS at a concentration of 250 mg/mL and loaded in the device in the same way as vasohibin-1. Eight SD rats were included in this experiment; 4 rats received the FD40 delivery device (FD40DD) and 4 rats received only pelletized FD40.

### Experiment 2: Immunohistochemistry after Device Implantation

Immunostaining for vasohibin-1was performed 2 weeks after device implantation. Twelve SD rats were used as follows (Table 1): 4 rats received vehicle (non-vasohibin-1) in the delivery device on the sclera (NVDD), 4 rats received 1.5 μL of 10 μM vasohibin-1 in the delivery device (10VDD), and 4 rats received 1.5 μL of 10 μM vasohibin-1 pellets implanted on the sclera. Immunohistochemistry was performed as reported previously [25].

Animals were euthanized using overdoses of ketamine hydrochloride and xylazine hydrochloride. The eyes were enucleated and fixed for 12 hours in 4% paraformaldehyde (PFA) at 4°C. The anterior segment and lens were removed from each eye. The posterior segment was cryoprotected at 4°C through successive 12-hour incubations in 10%, 20%, and 30% sucrose dissolved in saline. The tissues were immersed in OCT compound (Tissue-Tec; Sakura Finetec USA, Inc., Torrance, CA, USA) and frozen in acetone in a dry-ice bath. The frozen posterior segment was sectioned at the center of the implanted area at a thickness of 5 μm for each section, using a cryostat. We examined eight continuous sections per eye. The sections were incubated in rabbit polyclonal antibody against human vasohibin-1, followed by FITC-conjugated anti-rabbit IgG (1:200; Dako, Glostrup, Denmark) for 30 minutes. The sections were washed three times with PBS between each step. Negative controls (4 rats) incubated with just FITC-conjugated anti-rabbit IgG were also prepared. Slides were counterstained with 4, 6-diamino-1-phenylindole (DAPI; Vector Laboratories, Burlingame, CA, USA) and photographed using a fluorescence microscope (Leica FW4000, Ver. 1.2.1; Leica Microsystems Japan, Tokyo, Japan).

### Experiment 3: Choroidal Neovascularization Study

A total of 36 BN rats were used (Table 1). The devices and pellets were implanted on the same day as the CNV procedure. The rats were divided into six groups (6 rats in each group): rats with NVDD, rats with 1.5 μL of 1 μM vasohibin-1 in the delivery device (VDD), rats with 1.5 μL of 10 μM vasohibin-1 in the delivery device (10VDD), rats with 1.5 μL of 10 μM vasohibin-1 pellets implanted on the sclera, rats with intravitreal injection of 5 μL of vehicle, and rats with an intravitreal injection of 0.24 μM vasohibin-1 protein occurring 4 days after the experimental CNV procedure. The amount of intravitreal vasohibin-1 used and the day of the injection were determined based on our previous data [25]. The intravitreal injections were performed using a 10-μL glass syringe (Hamilton), and the needle was passed through the sclera just behind the limbus into the vitreous cavity.

#### 3 CNV procedure

A green argon laser was used to rupture the choroidal membrane using a slit-lamp delivery system (Ultima 2000SE; Lumenis, Yokneam, Israel) with a contact lens [31]. The laser settings were: 50 µm diameter for 0.1 sec duration, at an intensity of 650 to 750 mW. Six laser burns were made around the optic disc (Fig. 1A). Each burn was confirmed to have induced sub-retinal bubbles, indicating a rupture of Bruch's membrane.

In addition to the routine ophthalmological examinations, fluorescein angiography (FA) with an imaging system (GENESIS-Df; Kowa, Tokyo, Japan) was performed at 1 and 2 weeks after the CNV laser burn, and choroidal flat mounts of the CNV site were performed at 2 weeks after the procedure. Two retinal specialists (HO and TA) and one non-specialist (NN) evaluated the angiograms for FA grading evaluation in a blinded manner using a grading system [32], where Grade 1  =  no hyperfluorescence; Grade 2 =  hyperfluorescence without leakage; Grade 3 =  hyperfluorescence in the early or middle phase and leakage in the late phase; and Grade 4 =  bright hyperfluorescence in the transit and leakage in the late phase beyond the treated areas. The camera was a handheld retinal camera for photographing humans, and the fact that rat eye optics differ from that of humans made the process somewhat difficult. Intense fluorescein leakage also made the results of photographs as faint. The laser burn sometimes made subretinal hemorrhages that were shown as fluorescein blockage. These results may have influenced the evaluation. We tried to focus on the laser burn as much as possible to not influence the evaluation. Further we also tried to synchronize evaluations as much as possible to avoid significant bias due to fluorescein leakage. Total grades were analyzed for statistical significance.

#### 4 Fluorescein-Labeled Dextran Perfusion and Choroidal Flat-Mount Preparation

The size of the CNV lesion was measured on choroidal flat mounts to examine the effect of the vasohibin-1 delivery device (n = 6 eyes/group and each eye had 6 laser spots). Fourteen days after the CNV procedure, the rats were perfused with 5 mL PBS containing 50 mg/mL fluorescein-labeled dextran (FITC-dextran, MW: 2×10^6^; Sigma-Aldrich). Results of mouse CNV experiments [25] indicated that laser-induced CNV lesions were most active at 14 days after laser application and gradually self-resolved more than 28 days after the laser burn. This data was supported by our previous study of laser-burned monkey eyes [28].

We enucleated the eyes in the current study at 14 days after the CNV laser procedure, after euthanizing the animals per the previously described method. The eyes were removed and fixed for 30 minutes in 4% phosphate-buffered PFA. The cornea and lens were removed and the entire retina was carefully dissected from the eyecup. Radial cuts (4 to 6) were made from the edge to the equator, and the eyecup of the RPE-choroid-sclera (R-C-S) complex was flat mounted in Permalfuor (Beckman Coulter; Fullerton, CA, USA) with the scleral side facing down. Flat mounts were examined by fluorescence microscopy (Leica FW4000, Leica Microsystems Japan), and the total area of each CNV zone associated with each burn was measured. The CNV lesions were identified by the presence of fluorescent blood vessels on the choroidal/retinal interface circumscribed by a region lacking fluorescence. This process duplicated past reported procedures [33], [34]. Two retinal specialists (HO and TA) and one non-specialist (NN) evaluated the size of the dextran-fluorescein perfused CNVs in a blinded manner, as described above.

### Statistical Analyses

Analysis of variance (ANOVA) with Tukey's test was used to examine differences in the leakage and severity of the CNVs in the fluorescein angiograms and the area of the choroidal flat mount. Endothelial tube formation was also evaluated by this method. P-values less than 0.05 were considered significant.

## Results

### *In Vitro* Vasohibin-1 Release from the Device

Each result is shown as mean ± SD of three different experiments in Figure 1E. A prominent initial increase was observed in vasohibin-1 pellets (Pellet) and it appeared to almost plateau at 7 days after the start of incubation. A minor increase was observed in the vasohibin-1 delivery devices (VDD) with an almost level release observed over the 28 days of incubation. If we examine the amount released from the device (4×4×1.5 mm) between Days 7 and 28, the amount released was estimated to be 0.31 nM/day in the 10VDD group, 0.070 nM/day in the VDD group, 0.088 nM/day in the pellets, and 0 in the NVDD group (Fig. 1E) in a closed incubation system, when we used 500 mg/mL COLs for the permeable PEG/COLs membranes. These calculations were performed from the fitting line between 0 and 28 days. In rat experiments, the release amount would be less, because we used a smaller device for rats than used in the *in vitro* release assay. The larger device used in the *in vitro* release assay in Fig. 1E had 5.44 times (12.25 mm^2^ vs 2.25 mm^2^) larger drug-releasing surface area and 3.42 times faster releasing rate than that of the transplanted device used in rats, from the results of Fig. S1. The total amount of vasohibin-1 released from the 10VDD devices during the CNV suppression experiment in rats was estimated grossly to be approximately 4.28 nM over 2 weeks. The total amount of vasohibin-1 during the 2 weeks was estimated as about 14.6 nM from the results of Figure 1E, and was divided by 3.42, which is the difference in releasing rate between *in vitro* release assay and *in vivo* experiments, although the effective amount of vasohibin-1 in CNV suppression would be smaller than 4.28 nM, due to drug elimination from the eye. These results were confirmed by western blotting analysis; Figure 1D shows the representative results at Days 1, 7, 14, and 28. A greater amount of vasohibin-1 was observed in the 10VDD and pellet groups than was seen in the NVDD and VDD groups. The results of the pellet group at Day 1 (1d in Fig. 1D) was obtained after diluting the samples five times, because the concentration was too high to be shown by western blotting. However, the size of the pellets was much smaller after 7 days of incubation.

### Endothelial Tube Formation

**Figure 2:**
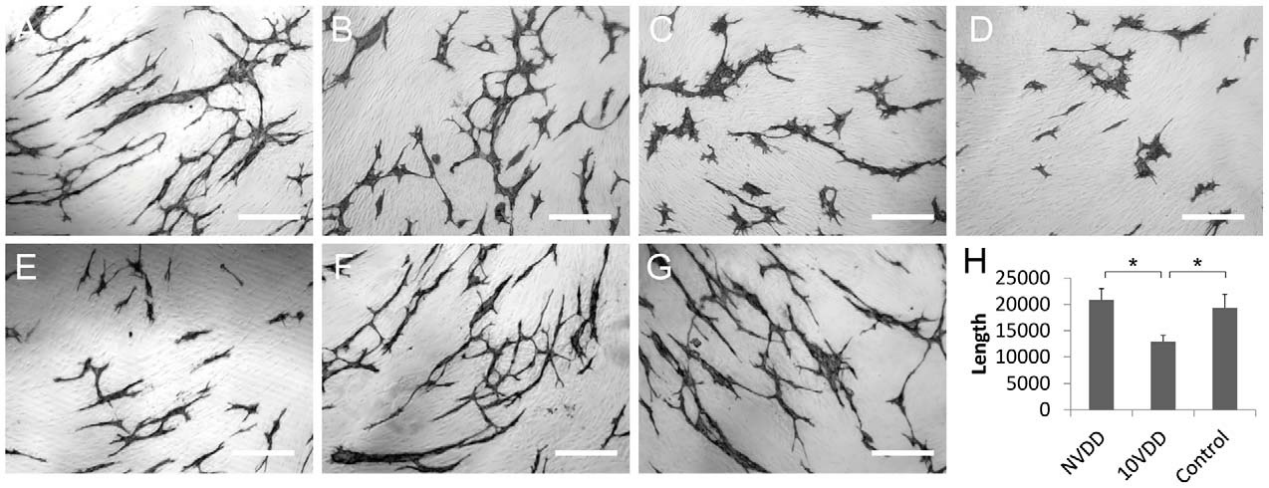
The activity of vasohibin-1 by an endothelial cell tube formation assay. The activity of vasohibin-1 was confirmed by an *in vitro* endothelial cell tube formation assay. Vasohibin-1 suppressed the HUVEC tube formation in a dose-dependent manner. Representative results of HUVEC tube formation treated with 2 nM VEGF combined with 0 (A), 0.2 (B), 2 (C), and 10 nM vasohibin-1 (D) are shown. Bars indicate 100 μm. The released vasohibin-1 from the device showed comparable results to native activity (E). Significant suppression of HUVEC tube formation was observed in released vasohibin-1 when compared to those treated with NVDD (F) and with only 2 nM VEGF without vasohibin-1 (G). (H) shows the average of each experiment; significantly fewer CD31-positive points were observed in released vasohibin-1-treated wells when compared to those of the vehicle released from NVDD (p<0.0001) or the VEGF-treated control (p<0.0001). The vasohibin-1 released from the device showed activity comparable to the native vasohibin-1. Vertical bar indicates total length of tube formation. NVDD: non-vasohibin-1 (vehicle) delivery device, 10VDD: 10 μM vasohibin-1 delivery device.

Endothelial tube formation of HUVECs cultured on the NHDF layer was assessed using anti-human CD31 immunostaining (Fig. 2). We used a range of native vasohibin-1 concentrations (from 0 to 10 nM, using 2 nM VEGF) for the preliminary experiments. After the initial examination, the cells were fixed and stained using anti-human CD31. Figures 2A–2G show representative photographs of the experimental results. Figure 2E shows the results of released vasohibin-1 (0.56 nM) from the devices with 2 nM VEGF. Figure 2H shows the average of each experiment; significantly fewer CD31-positive points were observed in released vasohibin-1-treated wells when compared to those of the vehicle released from the NVDD (p = 0.000001) or VEGF-treated control (p = 0.000002). Vasohibin-1 released from the device showed activity comparable to the native vasohibin-1.

### Macro Examination

FD40 was detected in the device (Figs. S2A and S2B show color and fluorescein photographs, respectively) or in pellets (Figs. S2G and S2H) at the implant site through the conjunctiva in the live rats. When we enucleated the eyes at a week after device implantation, mild fibrosis was observed around the devices (Fig. S2C) and around the pellets (Fig. S2I). Fluorescein photography demonstrated the presence of FD40 in the device, with little fluorescein in the conjunctiva and surrounding tissues (Fig. S2D, arrow). FD40 was also detected in the sclera after removal of the device (Figs. S2E and S2F, arrow). Conversely, FD40 pellets showed strong fluorescein on the conjunctiva and surrounding tissues, as was seen for the pellet itself (Fig. S2J, arrow). Furthermore, little fluorescein was observed on the sclera after removal of the device (Figs. S2K and S2L, arrow). Similar conditions were observed when we examined the tissues at 2 weeks after device and pellet implantation; fluorescence was observed over a wider area for those specimens where the device was implanted compared to results at Week 1 (data not shown).

### Immunohistology of Vasohibin-1

**Figure 3:**
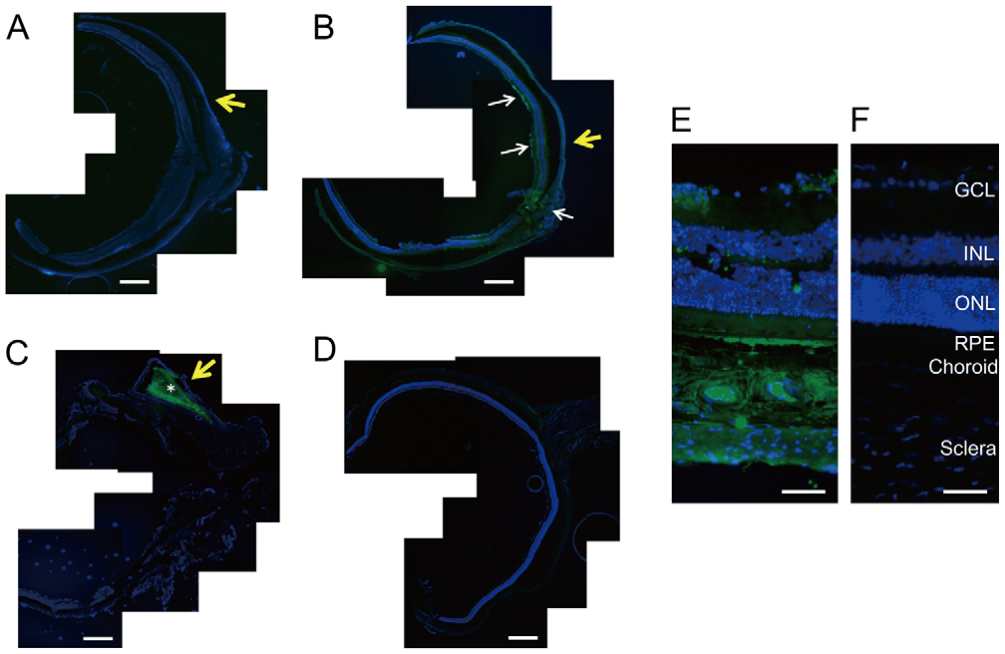
Immunohistochemistry of vasohibin-1 after device implantation. The immunohistochemistry results of vasohibin-1 after NVDD, 10VDD, and pellet implantation are shown. No immunoreactivity was observed after NVDD transplantation (A) and negative control without first antibody (D). 10VDD shows vasohibin-1 immunoreactivity at the device implant area (B). White arrows show the immunoreactivity in the retina and optic nerve at low magnification. Diffuse immunoreactivity was observed in the sclera, choroid, RPE, and retina at greater magnification (E). Strong immunoreactivity was observed in the ganglion cell layer (GCL) and retinal pigment epithelium (RPE), as well as in the sclera and choroid. INL and ONL indicate the inner and outer nuclear layers. These results were not observed in the NVDD group (A) or the negative controls (D and F). Strong immunoreactivity was observed in the pellet (asterisk) and in the tissues surrounding the implanted pellet (C). Yellow arrows indicate the positions where devices or pellets were placed. Devices were removed before sectioning, but pellets were not removed before sectioning. Bars: 200 μm (A–D), and 50 μm (E, F).

In immunostained eyes, vasohibin-1-positivity was found in only the 10VDD group (Fig. 3B), but not in the NVDD group (Fig. 3A) or the negative control without the first antibody (Fig. 3D), mainly at the region where vasohibin-1 releasing devices were placed. Pellets showed strong local immunoreactivity, but no immunoreactivity in the retina (Fig. 3C). Vasohibin-1 positivity was observed in the neural retina and optic nerve (white arrows in Fig. 3B). Strong immunoreactivity was observed in the choroid, RPE, and at the inner layer (such as the ganglion cell layer [GCL]) by magnified photographs after device implantation (Fig. 3E).

### Leakage from CNV

**Figure 4:**
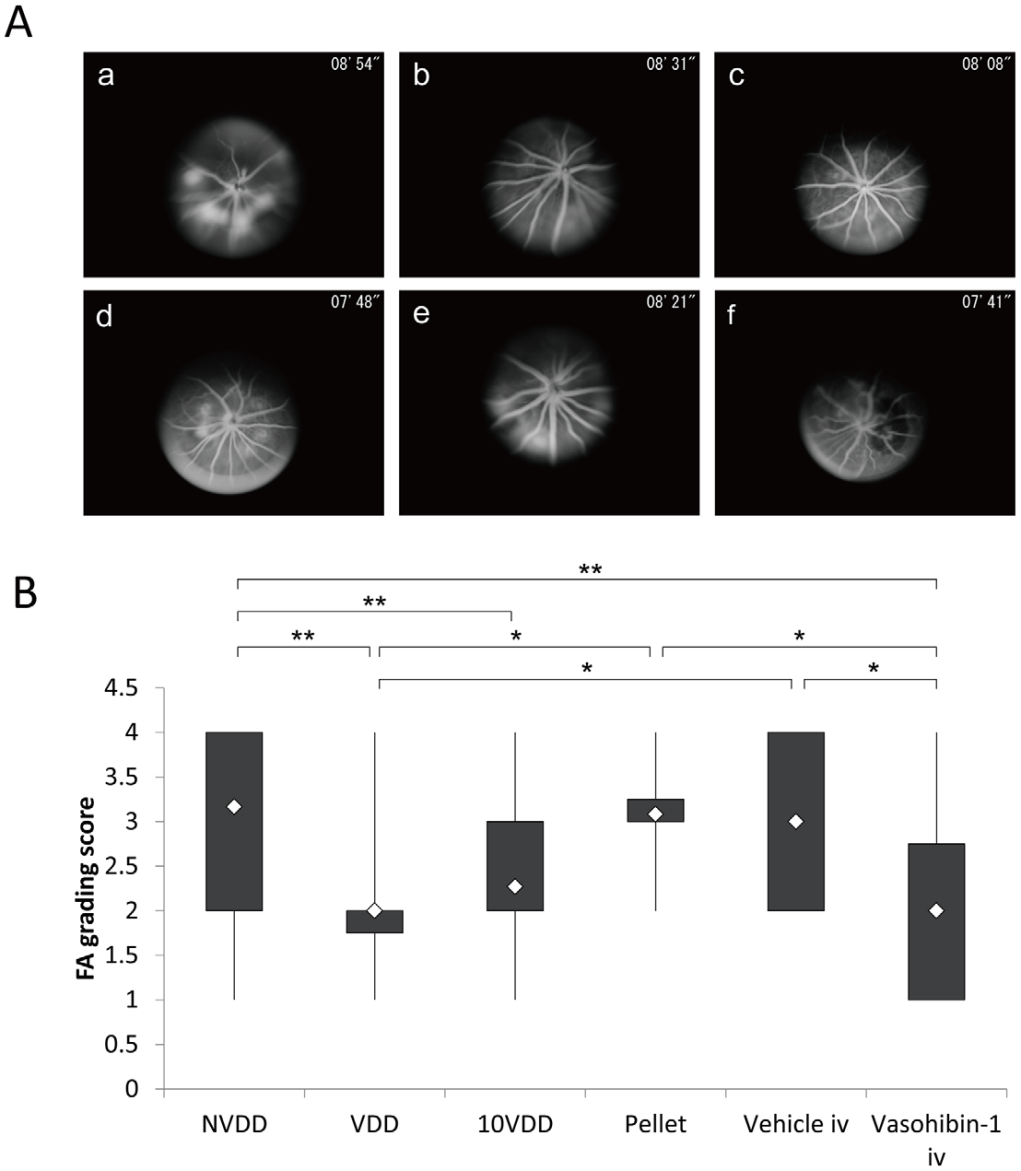
Fluorescein angiography 1 week after CNV laser procedure. (A) Representative results of fluorescein angiography (FA) in each group at 1 week after CNV laser procedure. The groups were treated with NVDD (a), VDD (b), 10VDD (c), vasohibin-1 pellet (d), intravitreal vehicle injection (Vehicle iv) (e), or intravitreal vasohibin-1 injection (Vasohibin-1 iv) (f). (B) Fluorescein angiography scores for each of the six laser spots in each eye are plotted and calculated for each group. Significantly lower FA scores was shown in the Vasohibin-1 iv group when compared to those of NVDD (p = 0.00014), pellet (p = 0.02), and Vehicle iv (p = 0.040). Significantly lower FA scores are also observed in the 10VDD group when compared to the NVDD group (p = 0.00006). Significantly lower FA scores are also observed in the VDD group when compared those of NVDD (p = 0.00017), Pellet (p = 0.012), and intravitreal vasohibin-1 injection (p = 0.026). Significant differences are shown as asterisks. NVDD: non-vasohibin-1 (vehicle) delivery device, VDD: 1 μM vasohibin-1 delivery device, 10VDD: 10 μM vasohibin-1 delivery device, Pellet: vasohibin-1 pelletized at the same concentration of 10VDD (without reservoir and cover).

**Figure 5:**
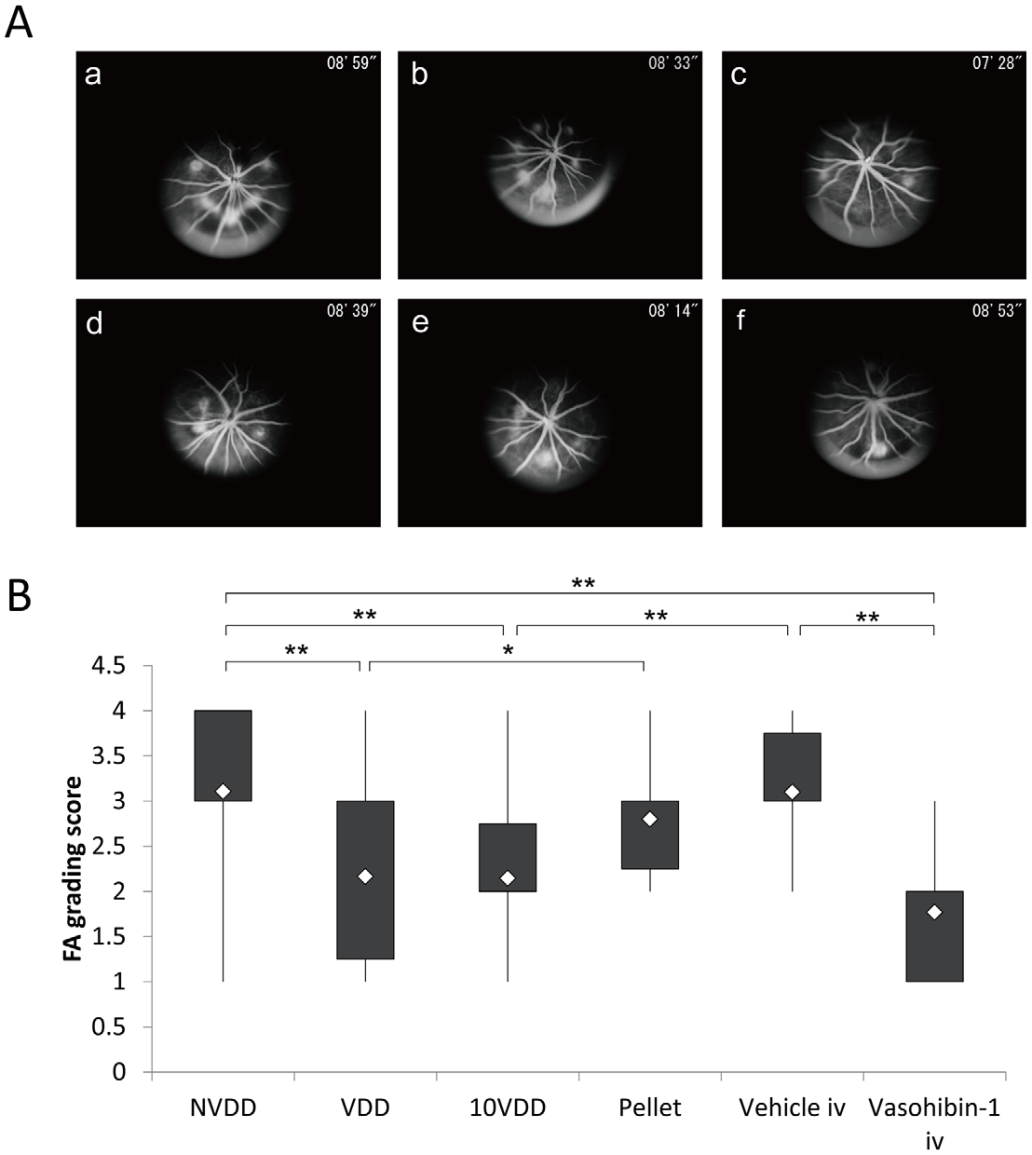
Fluorescein angiography 2 weeks after CNV laser procedure. (A) Representative results of fluorescein angiography in each group at 2 weeks after CNV laser procedure. The groups were treated with NVDD (a), VDD (b), 10VDD (c), vasohibin-1 pellet (d), intravitreal vehicle injection (Vehicle iv) (e), intravitreal vasohibin-1 injection (Vasohibin-1 iv) (f). (B) Significantly lower FA scores was shown in the Vasohibin-1 iv group when compared to those of NVDD (p = 0.000022), and Vehicle iv (p = 0.0065). Significantly lower FA scores are also observed in the 10VDD group when compared to the NVDD group (p = 0.00003) and intravitreal vehicle injection (p = 0.011). Significant differences are shown as asterisks. NVDD: non-vasohibin-1 (vehicle) delivery device, VDD: 1 μM vasohibin-1 delivery device, 10VDD: 10 μM vasohibin-1 delivery device, Pellets: vasohibin-1 pelletized at the same concentration of 10VDD (without reservoir and cover).

Fluorescein angiography results of each group at 1 week after the laser CNV procedure are shown in Figure 4A. The results show that an intravitreal injection of vasohibin-1 on Day 4 after the CNV procedure led to a significant reduction of FA scores when compared to those of NVDD (p = 0.00014), pellet (p = 0.020), and vehicle injection (p = 0.040) (Fig. 4B). The 10VDD implantation led to a significant reduction of FA scores when compared to the result of the NVDD group (p = 0.00006). The VDD implantation led to a significant reduction of FA scores when compared to those of NVDD (p = 0.000017), pellet (p = 0.012), and vehicle injection (p = 0.026). Although FA scores of the 10VDD group seemed to be smaller than those of the pellet (p = 0.065) and vehicle injection (p = 0.12), the results were not significant. Figure 5A shows the FA results at Week 2 in each group. Significantly lower FA scores were observed for the vasohibin-1 intravitreal injection group when compared to those of NVDD (p = 0.000022), and vehicle intravitreal injection (p = 0.0065). Further, significantly lower FA scores were observed in the 10VDD group when compared to those of NVDD (p = 0.000003) and vehicle injection (p = 0.0080) (Fig. 5B). Significantly lower FA scores were also observed in the VDD group when compared to those of NVDD (p = 0.000058) and vehicle injection (p = 0.011).

### Flat-mount Examination of the CNV Site

**Figure 6:**
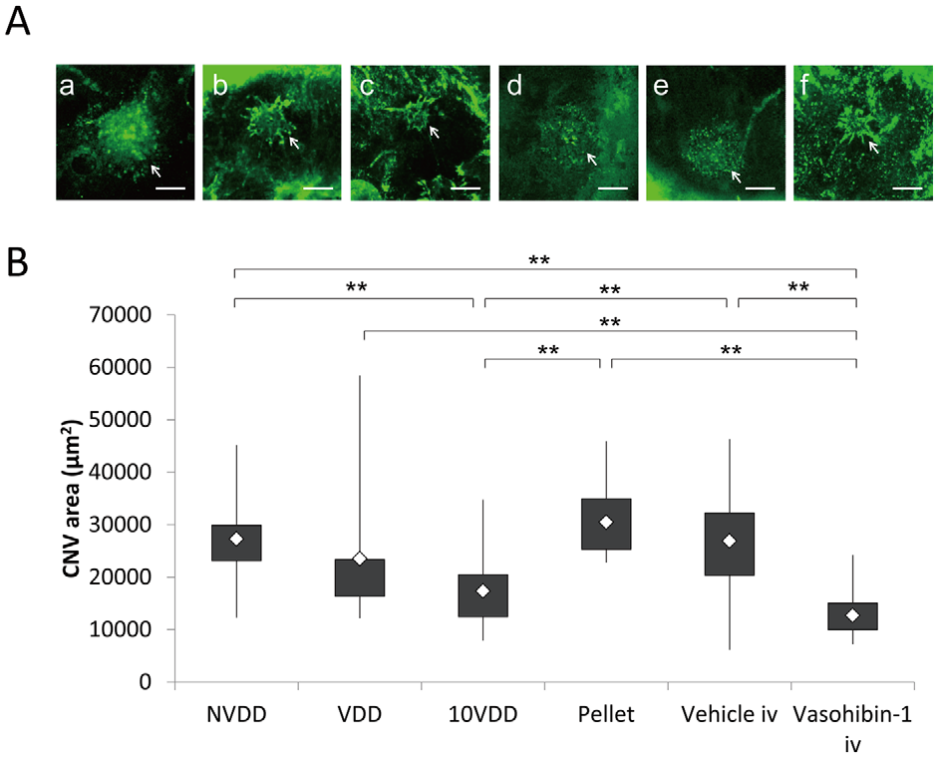
Flat-mount examination of the CNV site. The areas of choroidal neovascularization with devices, pellets, and intravitreal injection of recombinant vasohibin-1 protein. (A) Representative choroidal flat-mount photographs of the groups treated with NVDD (a), VDD (b), 10VDD (c), vasohibin-1 pellet (Pellet) (d), intravitreal vehicle injection (Vehicle iv) (e), intravitreal vasohibin-1 injection (Vasohibin-1 iv) (f) eyes at 2 weeks after the CNV laser procedure. Mean values of actual areas are shown in the text. Bars: 200 μm. (B) Significantly smaller CNV areas were observed in the 10VDD group when compared to those of the NVDD (p = 0.0004), Pellet (p = 0.0011), and Vehicle iv groups (p = 0.000015). Significantly smaller CNV areas were observed in eyes treated with Vasohibin-1 iv when compared to those treated with NVDD (p = 0.000006), VDD (p = 0.0036), Pellet (p = 0.000023), or Vehicle iv (p = 0.000001). NVDD: non-vasohibin-1 (vehicle) delivery device, VDD: 1 μM vasohibin-1 delivery device, 10VDD: 10 μM vasohibin-1 delivery device, Pellet: vasohibin-1 pelletized at the same concentration of 10VDD (without reservoir and cover).

Choroidal flat mounts were prepared 2 weeks after device implantation; representative results of each group are shown in Figure 6A. The area of the CNV was 27,288±7,975 µm^2^ for the NVDD group; 23,532±13,120 µm^2^ for the VDD group; 17,382±715 µm^2^ for the 10VDD group; 30,502±780 µm^2^ for the vasohibin-pellet group; 26,900±9,067 µm^2^ for the intravitreal vehicle injection group, and 12,731±4,113 µm^2^ for the intravitreal vasohibin-1 injection group (Fig. 6B). The CNV area was smaller in eyes that were treated with 10VDD or intravitreal vasohibin-1 injection compared to the other treatments. A significantly smaller CNV area was observed in the 10VDD group when compared to those of the NVDD (p = 0.0004), pellet transplantation (p = 0.0011), and intravitreal vehicle injection groups (p = 0.000015). A significantly smaller CNV area was also observed in eyes injected with intravitreal vasohibin-1 when compared to those of the NVDD (p = 0.000006), VDD (p = 0.0036), pellet transplantation (p = 0.000023), and intravitreal vehicle injection groups (p = 0.000001) (Fig. 6B). No significant difference was observed when we compared the VDD with those of NVDD (p = 0.7374), pellet transplantation (p = 0.3616), and intravitreal vehicle injection (p = 0.7178) groups.

## Discussion

Attention has been paid to sustained drug delivery in the treatment of AMD because regimens including intravitreal anti-VEGF injection require repeated injection and may lead to adverse side effects [9], [35]. Sustained delivery of large molecules such as antibodies may be attractive, because not only anti-VEGF therapy and anti-TNFα antibody have shown excellent results in the treatment of refractory eye diseases (such as Behcet's disease), although this regimen also requires repeated cycles of therapy [36], [37]. When our devices were cultured in PBS, vasohibin-1 was released over time, with activity equivalent to that seen with native vasohibin-1. These positive results were also observed with brain-derived neurotrophic factor (BDNF) and 40 kDa dextran, as reported previously [24]. Our implantable device showed sustained protein release over time. The relatively large standard deviation in the 10VDD group may be indicative of imperfect device preparation. From the results of western blotting, the 10VDD group showed a mild initial release of drug, although the level was far less than seen in the pellet-only group. Technical improvements in delivery device design may overcome these problems. This is an attractive device designed with sustained protein delivery for the treatment of eye diseases.

Subconjunctival drug administration produces better drug penetration than eye drops and is less invasive than intravitreal injection. However, conjunctival and episcleral blood and lymphatic flows have been reported to be the main limiting factors for posterior segment drug distribution by subconjunctival drug administration [38]–[40]. Our results also showed that implantation of pelletized vasohibin-1 alone (with no reservoir) produced much less vasohibin-1 immunoreactivity than seen with 10VDD implantation. Implanted between the sclera and conjunctiva, our device was designed to release the drugs only to the scleral side of the eye, so a limiting factor of drug diversion to the conjunctival blood flow may be reduced. Carvalho et al [41] reported that their tightly-sutured, one-side-open device delivered higher amount of sodium fluorescein than others, although they used small molecules with their device. From the histological analysis of our experimental procedure, we saw no signs of inflammation or adverse effects in the eye that could be attributed to device implantation, except for a mild fibrosis observed around the devices at 2 weeks post-surgery. We also found that the devices removed from the rats where fibrosis was noted showed continuing vasohibin-1 release and comparable activity when we cultured the removed device/tissues in PBS (data not shown).

Vasohibin-1 was observed on the retina at 2 weeks post-implant, principally noted in the regions where the devices were implanted. Some of the regions showed strong immunoreactivity for vasohibin-1, especially at the retinal pigmented epithelium (RPE) and the retinal ganglion cell layer (GCL); the first finding may be due to being the main outer blood-retinal barrier, while the second may be due to the vitreous-retinal barrier [42]. Vasohibin-1 released from the device may be stored in cells in these regions and later released to other regions of the retina or vitreous.

Our results demonstrated that vasohibin-1 can be delivered by our device into the retina transsclerally. Amaral et al also reported transscleral protein (pigment epithelium-derived factor and ovalbumin) delivery into the retina, although they used uncontrollable drug release via a matrix-type implant [10]. Drug released from their device was not delivered unidirectionally. Although there is a blood-retinal barrier, the penetration of such large molecules into the eye may not be so surprising. When we consider the phenomenon of some type of cancer-associated retinopathy, auto-antibodies against retinal cells or retinal-specific antigens have been reported to cause retinal dysfunction [43]–[45]. The elimination of proteins is reported to be one to two orders of magnitude slower than that of small molecules via the subconjunctival and episcleral blood flow [46], with similar results reported for the choroidal blood flow [21]. This fact may also help protein delivery to the retina with the use of our device.

Although we have not studied vasohibin-1 release from the device for more than 2 weeks *in vivo* because of the experimental design, more than 80% of the vasohibin-1 was present in the device at the end of the experimental procedure. The devices removed at the end of the experiment were still releasing vasohibin-1 (data not shown), indicating that it might be possible to use the implanted device for a longer time. These data could also indicate that we may be able to use a smaller device than those used in this experiment to deliver the same amount of drug.

Fluorescein angiography examination showed significantly lower scores in the eyes that received intravitreal vasohibin-1 than those of the intravitreal vehicle-injected eyes. The effects of vasohibin-1 were also confirmed from the flat-mount experiments. These results were same as those previously noted in mice [25]. Our 10VDD device delivered vasohibin-1 to the retina transsclerally, with results comparable to those seen with intravitreal vasohibin-1 injections. With a less invasive method than that of intravitreal injection and the added advantage of continuous drug delivery, our device may be able to replace invasive intravitreal drug injections. Although there was no significant difference between 10VDD and VDD when we evaluated by FA, a statistically significant effect was observed in only 10VDD, but not VDD when we performed the flat-mount examination. One of the reasons these two do not match exactly may be due to the uncertainty about the FA evaluation, as not only blockage by hemorrhage, but also tissue staining and/or leakage sometimes make evaluation difficult [47]. Further study is needed to determine the exact amounts of vasohibin-1 released from the device, the kinetics of drug distribution, the correlation between drug amount and ocular distribution, and the effects of this regimen on CNV, as well as the appropriate duration of vasohibin-1 release.

Choroidal neovascularization has been reported to be produced by choriocapillaris of the choroidal blood flow [48]. Many effects of choroidal blood flow or RPE may stimulate CNV formation into the retina [49]. Drusen, a preclinical feature of age-related macular degeneration, also stimulates CNV formation [50]. Transscleral anti-CNV drug delivery will be more reasonable than that of intravitreal injection not only from the points of safety, but also from the aspect of CNV pathophysiology. The RPE and RPE-choroid complex are reported to be one to two orders of magnitude slower in drug penetration [21]. When we put our device on the sclera, the drug can pass through the sclera and reach the choroid and RPE earlier than the retina. Between the choroid and neural retina, anti-CNV drugs released from our device may suppress on-going CNV formation. Suprachoroidal bevacizumab was reported to be delivered to the RPE, choroid, and photoreceptors, whereas intravitreal injection distributed more to the inner retina [11]. Olsen et al stressed the importance of delivery of a sustained-release formulation of large molecules to the suprachoroidal space [11]. Our device will offer a safer therapeutic method than those previously reported, especially in the treatment of AMD.

### Conclusion

We developed a sustained delivery device for the release of vasohibin-1 in the eye. The released vasohibin-1 showed activity comparable to vasohibin-1 delivered via other methods. When we placed the device on the rat sclera, we found vasohibin-1 released to the sclera, retinal pigment epithelium, and retina. Transscleral vasohibin-1 delivery significantly reduced laser-induced CNV that are comparable as those of effects seen with intravitreal vasohibin-1 injection in the rat eye. Our device will offer a safer therapeutic method than intravitreal injections.

## Supporting Information

Figure S1 **The size of the devices.** The size of the device was 4 mm×4 mm×1.5 mm for the vasohibin-1 releasing assay (A, Device (a)) and 2 mm×2 mm wide ×1 mm high for the rat experiments (A, Device (b)). Because it was very difficult to detect using standard ELISA techniques, we used a larger size device for ELISA. The vasohibin-1 releasing area was 5.44 times larger in Device (a) (3.5 mm×3.5 mm  = 12.25 mm^2^) than that of Device (b) (1.5 mm×1.5 mm  = 2.25 mm^2^). Bar: 5 mm. We formulated fluorescein isothiocyanate (FITC) dextran (FD40) as simulated drugs and the device was incubated in a Transwell in 400 μL of PBS at 37°C. To estimate the amounts of FD40 that had diffused out of the Transwells, the fluorescent intensities of the PBS solutions were measured spectrofluorometrically (FluoroscanAscent; Thermo). From the results of a fitting curve (B), we calculated that the releasing rate of the larger device was 0.958 μg/hr/day, whereas the smaller device released 0.28 μg/hr/day; the difference of the releasing rates was calculated as 3.42 (0.958/0.28).
(TIF)

Figure S2 **Comparison of FD40DD and FD40 pellet implantations.** Rats implanted with FD40DD or FD40 pellets are shown. Devices or pellets were confirmed by color photographs (A and G), after enucleation (C and I), and after device (E) or pellet (K) removal. Mild fibrosis was observed around the devices (C) or pellets (I). FD40 was detected in the device (B), or pellets (H) by fluorescein photography at the site of the implant through the conjunctiva in the live rats during the experiment. When the eyes were enucleated at 1 week after device implantation, little fluorescence was observed in the conjunctiva and surrounding tissues (D, white arrow) in FD40DD-treated rats, whereas strong fluorescence in the conjunctiva was observed in pellet-treated rats (J, white arrow). FD40 was also detected on the sclera after removal of the device (F), but not the pellet (L) (yellow squares indicate the implantation site).
(TIF)
