# Supplementary figures and images for: Transscleral Sustained Vasohibin-1 Delivery by a Novel Device Suppressed Experimentally-Induced Choroidal Neovascularization
Source: PLoS One. 2013 Mar 5;8(3):e58580. doi: 10.1371/journal.pone.0058580 (PMC3589385; doi:10.1371/journal.pone.0058580)

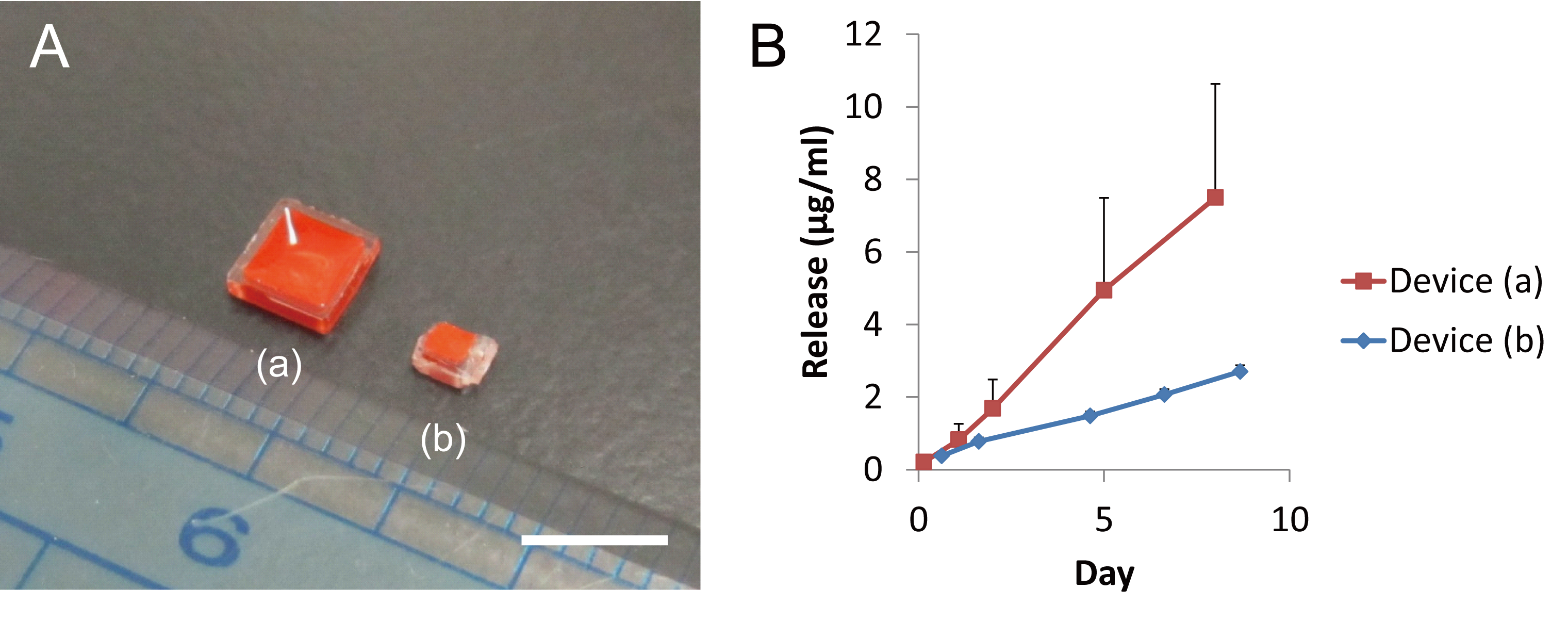

Supplement: Figure S1 — The size of the devices. The size of the device was 4 mm×4 mm×1.5 mm for the vasohibin-1 releasing assay (A, Device (a)) and 2 mm×2 mm wide ×1 mm high for the rat experiments (A, Device (b)). Because it was very difficult to detect using standard ELISA techniques, we used a larger size device for ELISA. The vasohibin-1 releasing area was 5.44 times larger in Device (a) (3.5 mm×3.5 mm = 12.25 mm2) than that of Device (b) (1.5 mm×1.5 mm = 2.25 mm2). Bar: 5 mm. We formulated fluorescein isothiocyanate (FITC) dextran (FD40) as simulated drugs and the device was incubated in a Transwell in 400 μL of PBS at 37°C. To estimate the amounts of FD40 that had diffused out of the Transwells, the fluorescent intensities of the PBS solutions were measured spectrofluorometrically (FluoroscanAscent; Thermo). From the results of a fitting curve (B), we calculated that the releasing rate of the larger device was 0.958 μg/hr/day, whereas the smaller device released 0.28 μg/hr/day; the difference of the releasing rates was calculated as 3.42 (0.958/0.28). (TIF) [file pone.0058580.s001.tif]

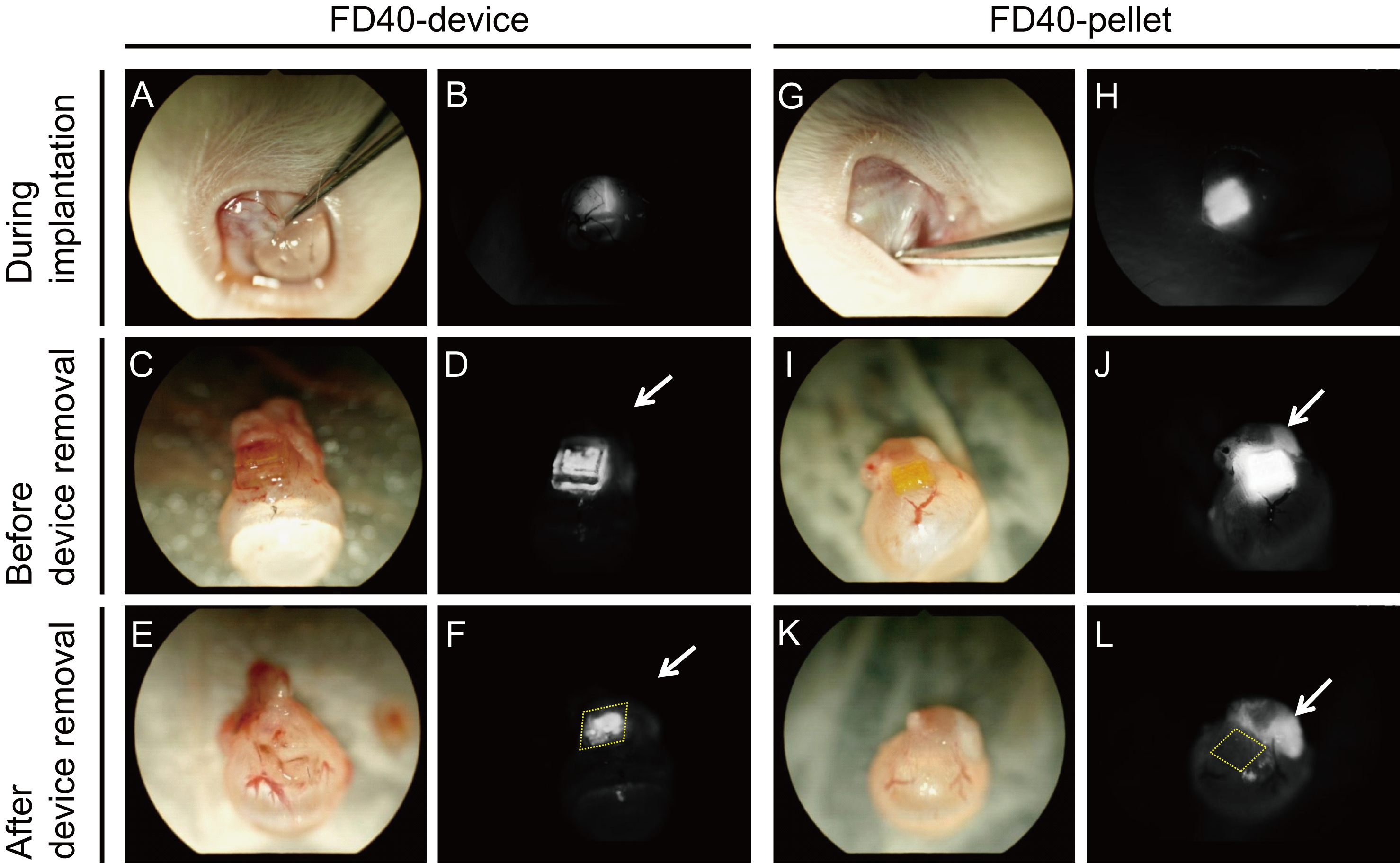

Supplement: Figure S2 — Comparison of FD40DD and FD40 pellet implantations. Rats implanted with FD40DD or FD40 pellets are shown. Devices or pellets were confirmed by color photographs (A and G), after enucleation (C and I), and after device (E) or pellet (K) removal. Mild fibrosis was observed around the devices (C) or pellets (I). FD40 was detected in the device (B), or pellets (H) by fluorescein photography at the site of the implant through the conjunctiva in the live rats during the experiment. When the eyes were enucleated at 1 week after device implantation, little fluorescence was observed in the conjunctiva and surrounding tissues (D, white arrow) in FD40DD-treated rats, whereas strong fluorescence in the conjunctiva was observed in pellet-treated rats (J, white arrow). FD40 was also detected on the sclera after removal of the device (F), but not the pellet (L) (yellow squares indicate the implantation site). (TIF) [file pone.0058580.s002.tif]
